# Burden and correlates of atrial fibrillation among hypertensive patients attending a tertiary hospital in Tanzania

**DOI:** 10.1186/s12872-020-01517-x

**Published:** 2020-05-19

**Authors:** Smita Bhalia, Pedro Pallangyo, Abuu Dalidali, Saada Salum, Richard Kawajika, Edna Kajuna, Happiness Kusiima, Engerasiya Kifai, Peter Kisenge, Tatizo Waane, Mohamed Janabi

**Affiliations:** 1 Department of Adult Cardiology, Jakaya Kikwete Cardiac Institute, P. O Box 65141, Dar es Salaam, Tanzania; 2 Department of Clinical Support Services, Jakaya Kikwete Cardiac Institute, P. O Box 65141, Dar es Salaam, Tanzania; 3 Department of Nursing, Jakaya Kikwete Cardiac Institute, P. O Box 65141, Dar es Salaam, Tanzania

**Keywords:** AF, Hypertension, Tanzania

## Abstract

**Background:**

Atrial fibrillation (AF) is the most common supra ventricular cardiac arrhythmia, which presents with variety of clinical symptoms. Hypertension increases risk of developing Atrial fibrillation by 1.5 fold. Together Atrial fibrillation and hypertension doubles the risk of morbidity and mortality. We aimed to determine the prevalence of AF and describe associated factors among hypertensive patients attending tertiary hospital in Tanzania.

**Methods:**

A cross-sectional hospital-based study, involving 391 hypertensive patients visiting the Jakaya Kikwete Cardiac Institute was conducted between October to December 2017. Categorical variables were analyzed using chi square while student t- test was used to analyze continuous variables. Multivariate logistic regression analysis was performed to determine factors associated with AF. All analysis was two sided and p- value of < 0.05 was used to be not significant.

**Results:**

AF was detected in 40 (10.2%) patients. Atrial fibrillation was associated with BMI ≥ 25 (OR 4.4, 95% CI 1.1–7.7, *p*-value 0.02), ejection fraction < 50% (OR 3.0, 95%CI 1.1–8.2, p-value 0.03), Left Atrial diameter > 40 mm (OR 9.4,95%CI 2.1–43.2, p-value < 0.01) and eGFR< 60 (OR 2.9, 95%CI 1.1–7.8, p-value 0.04).

**Conclusion:**

Atrial fibrillation is considerably prevalent among the hypertensive sub-population. Prompt diagnosis and timely management is vital to prevent complications and premature mortality.

## Background

Hypertension is on the rise in low and middle-income countries who historically have battled communicable diseases. European society of cardiology (ESC) in 2016 reported that there were 20.9 million men and 12.6 million females living with atrial fibrillation around the world [1]. Health facilities in Tanzania are now faced with an ever growing number of hypertensive patients with multiple comorbidities including atrial fibrillation. There is a five-fold increase of atrial fibrillation in the presence of hypertension and hypertension related heart diseases [2]. The link between hypertension and atrial fibrillation is unclear. Timely diagnosis of Atrial fibrillation and maintaining blood pressure at optimal levels have been shown to significantly lower morbidity and mortality among hypertensive patients. This situation calls for keener clinical assessment of hypertensive patients for timely diagnosis and management of AF as well as maintenance of blood pressure at target levels.

## Methodology

### Recruitment

A total of 491 hypertensive patients were consecutively enrolled in this Cross-sectional hospital-based study conducted at Jakaya Kikwtete Cardiac Institute. For the purpose of this study and after thorough literature review we developed and pre tested a questionnaire, comprising of sociodemographic, and clinical history followed by anthropometric measurement and 12 lead ECG testing using Phillips machine, echocardiography using Siemens SC300 and blood test results of lipid profile, serum creatinine and random blood sugar. Anthropometric measurement and ECG were performed by trained medical assistant and echocardiography were performed by blinded senior cardiologist.

### Statistical analysis

Data analysis was done using SPSS (Statistical package for social sciences) version 20 Categorical variables were analyzed using chi square while student t- test was used to analyze continuous variables. Multivariate logistic regression analysis was performed to determine factors associated with AF. All analysis was two sided and p- value of < 0.05 was used to be not significant.

## Results

A total of 391 hypertensive patients were enrolled into the study during Oct – Dec 2017. Mean age of participants was 58 ± 13.47 years, 56% were male, 53.7% used alcohol and 11.3% smoked. Mean SBP, DBP and pulse rate were 150.1 ± 28.1, 85.7 ± 18.6 and 81.8 ± 20.3 respectively. 79.8% of the participants were on anti-hypertensive medication, 28.1% had renal insufficiency and 58.3% had anemia. General characteristics of participants are summarized in the Table 1.

**Table 1:** The baseline characteristics of study population at JKCI (N-391)

Characteristics	
**Sociodemographic**	
Age mean (SD)	58.01 (13.5)
Sex; Male (%)	221 (56.5)
Female (%)	170 (43.5)
Marital status; Married/ Cohabiting (%)	301 (77)
Single/divorced/ widow/widower (%)	90 (23)
Education; Formal (%)	326 (83.4)
Informal (%)	65 (16.6)
Alcohol (%)	210 (53.7)
Smoking (%)	44 (11.3)
**Clinical characteristics**	No (%)
Chest pain	85 (21.7)
Easy fatigability	93 (237)
Cough	37 (9.5)
Awareness of heartbeat	56 (14.3)
Shortness of breath	121 (30.9)
Body swelling	44 (11.2)
Cerebral vascular event	24 (6.1)
Others^a^	121 (30.9)
Diabetes Mellitus	92 (23.5)
Antihypertensive medication use	312 (79.8)
Weight mean (SD) in kg	75.41 ± 14.5
BMI	28.75 ± 5.3
SBP mean (SD) mmHg	150.12 ± 28.17
DBP mean (SD) mmHg	85.71 ± 18.60
Pulse	81.86 ± 20.32
**Lab characteristics**	mean ± SD
Creatinine umol/l	185.41 ± 289
eGFR(ml/min/1.73 m2)	77.88 ± 36.35
Total cholesterol (mmol/l)	5.02 ± 1.5
Triglyceride (mmol/l)	1.7 ± 0.88
HDL (mmol/l)	1.08 ± 0.34
LDL (mmol/l)	3.3 ± 1.3
Anemia	228 (58.3%)
**ECG**	
**AF**	**40 (10.23%)**
**No AF**	**351 (89.77%)**
**Echo (*N*** **= 265)**	
IVSD	14.31 ± 3.4
≥ 12 mm	184 (70%)
LVEF (%)	56.6 ± 18.8
LAD	56.5 ± 18.8

Continuous variables are presented as mean ± standard deviation, while categorical variables are presented as proportions, number of patients, Height and weight were used to calculate body mass index (*BMI* weight in kg /height in meters^2^), *SBP* systolic blood pressure, *DBP* diastolic blood pressure, *eGFR* estimated glomerular filtration rate calculated using modification of diet in renal disease study equation, *HDL* high density lipoprotein, *LDL* low density lipoprotein, *IVSD* interventricular septal diameter, *LVEF* left ventricular ejection fraction, *LAD* left atrial diameter. ^a^Other clinical presentation included: nausea, vomiting, fainting drug refill, follow-up clinic and referral from other clinic for investigation

### The sociodemographic characteristics associated with AF among hypertensive patients at JKCI

AF was detected in 40 (10.23%) patients. The sociodemographic characteristics in both patients with AF and without AF group were similar and the prevalence doubled from the age 58 years and above, alcohol consumption 189 (55.2%) and smoking 38 (12%) as shown in Table 2.

**Table 2:** Showing sociodemographic characteristics associated with AF among hypertensive patients (*N* = 391)

Characteristics		AF (%) (*N* = 40)	NO AF (%) (*N* = 351)	*P* value
Age (years)	< 65	22 (55)	242 (68.9)	0.074
	≥ 65	18 (45)	109 (31.1)	
Sex	Male	23 (57.5)	198 (56.4)	0.895
Education level	Formal education	32 (80)	294 (83.8)	0.507
Alcohol	Ever used	17 (42.5)	189 (53.8)	0.172
Smoking	Ever smoked	3 (7.3)	41 (11.7)	0.428

### Clinical characteristics associated with atrial fibrillation among hypertensive patients at JKCI. (N-391)

The most common clinical presentation of participants with AF were easy fatigability (42.5%) and palpitation (35%) and both were statistically significant compared to those without AF. Similarly, majority of the participants with AF had reduced ejection fraction < 50% (adjusted odds ratio 4.371, *p*-value 0.021, 95% CI 1.077–7.820) and dilated left atrial size ≥40 mm (adjusted odds ratio 9.417, p-value 0.004, 95%CI 2.051–43.247) and both were found to be independent predictors of AF. Left ventricular hypertrophy was assessed on ECG and ECHO. On ECG using Sokolow- Lyon criteria 30.8% (OR 2.05, p-value 0.044, 95%CI 1.019–4.008) while on echocardiography 57.7% of the participants with AF and 70.1% of the participants without AF had LVH as shown in Table 3.

**Table 3:** Clinical presentation of patients with atrial fibrillation among Hypertensive patients (*N* = 391)

Presenting complain	AF ***N*** = 40(%)	NO AF ***N*** = 351(%)	***P*** value
Chest pain	11 (27.5)	74 (21.1)	0.351
Shortness of breath	16 **(40)**	105 **(29.9)**	0.165
Palpitation	14 **(35)**	42 (12)	< 0.001
Easy fatigability	17 **(42.5)**	76 (21.7)	**0.003**
Body swelling	7 (17.5)	37 (10.5)	0.187
Cough	6 (15)	31 (8.8)	0.207
Stroke	4 (10)	20 (5.7)	0.856
Others*	4 (10)	117 (33.3)	0.002
DM	5 (12.5)	87 (24.8)	0.83
SBP ≥140 mmHg	21 (52.5)	244 **(69.5)**	**0.029**
DBP ≥90 mmHg	11 (27.5)	202 (57.5)	**0.068**
Heart rate			
≥ 100b/m	8 (20)	48 (13.7)	0.189
99-60b/m	25 (62.5)	265 (75.5)	0.087
< 59b/m	7 (17.5)	37 (10.5)	0.279
**Biochemical characteristics**			
Total Cholesterol	1.45 ± 0.5	1.47 ± 0.5	
≥ 5.2 mmol/l	18 (45)	174 (49.6)	0.584
< 5.2 mmol/l	22 (55)	177 (50.4)	
Triglyceride	1.61 ± 0.8	1.7 ± 0.88	
< 1.69 mmol/l	27 (67.5)	214 (61)	0.421
≥ 1.69 mmol/l	13 (32.5)	137 (39)	
HDL	1.00 ± 2.92	1.08 ± 0.3	
≥ 1.04 mmol/l	16 (40)	174 (49.6)	0.251
< 1.039 mmol/l	24 (60)	177 (50.4)	
LDL	3.2 ± 1.26	3.3 ± 1.3	
< 3.39 mmol/l	17 (42.5)	190 (54.1)	0.163
≥ 3.4 mmol/l	23 (57.5)	161 (45.9)	
BMI(N-377)	29.53 ± 5.25	28.82 ± 5.21	
≥ 25	30 (83.3)	228 (66.9)	**0.043**
Egfr	65.95 ± 31.20	79.24 ± 36.3	
< 60	17 (42.5)	93 (26.5)	0.033
**ECG**			
LVH (Sokolow)	14 (35)	73 (20.8)	**0.041**
**Echo*N*** **= 263**			
EF ≥50	8 (30.8)	171 (69.5)	**< 0.001**
< 50	18 (69.2)	75 (30.5)	
IVSD < 12 mm	11 (42.3)	72 (29.9)	**0.193**
≥ 12 mm	15 (57.7)	169 (70.1)	
LAD< 40 mm	2 (8)	129 (54)	**< 0.001**
≥ 40 mm	23 (92)	110 (46)	

*represents fever and diarrhea

*SBP* systolic blood pressure, *DBP* diastolic blood pressure, Cholesterol > 5.2 mmol/l was raised, triglyceride > 1.69 mmol/l raised, hdl < 1.04 mmol/l low, ldl 3.4 mmol//l raised were identified as dyslipidemia. Reduced ejection fraction was defined as EF < 50%, normal ≥50%, LVH on echocardiography was seen if IVSD was ≥12 mm, dilated left atrial diameter > 40 mm

Logistic regression model of 15 variables was used to assess for AF associated factors. in the bivariate model out of which nine variables were found to increase probability of developing AF, however, when those nine characteristics were adjusted for confounders in a multivariate model, four characteristics i.e. (BMI ≥25, EF < 50%, LAD > 40 mm and eGFR < 60 ml/min/1.73m^2^ were found to be independent predictors of AF as shown in Table 4 below.

**Table 4:** Logistic Regression analysis to determine factors associated with AF

Variable	Odds ratio	*P* value	95% CI	Adjusted odd ratio	95%CI	*P* value
Age ≥ 65 yr	1.817	0.077	0.936–3.524	–		
Male	1.045	0.895	0.540–2.026	–		
HDL ≥1.04 mmol/l	1.475	0.253	0.757–2.871	–		
LDL < 3.4 mmol/l	1.597	0.165	0.824–3.093	–		
SBP > 140 mmHg	0.485	0.032	0.250–0.939	–		
DBP > 90 mmHg	0.514	0.072	0.249–1.062	–		
Alcohol use	0.605	0.1312	0.312–1.172	–		
Cigarette Smoking	0.613	0.432	0.181–2.078			
Diabetes Mellitus	0.246	0.022	0.074–0.818	–		
BMI ≥ 25	2.478	0.049	1.002–6.126	**4.371**	1.077–7.820	0.021
EF < 50%	5.130	< 0.001	2.136–12.318	**3.013**	1.106–8.21	0.031
LAD > 40 mm	13.486	0.001	3.110–58.487	**9.417**	2.051–43.247	0.004
IVSD	0.581	0.197	0.254–1.326	**–**		
LVH on ECG	2.051	0.044	1.019–4.008	–		
eGFR < 60 ml/min/1.73m^2^	2.050	0.036	1.049–4.008	**2.902**	0.035	1.077–7.820

## Discussion

This study showed that the prevalence of AF in the patients visiting JKCI with hypertension is 10.23%. Similar results were observed previously in both western and Asian populations, (9.75%) Kosovo, (9.1%) Turkey and (8%) Brazil and slightly lower in studies conducted in Senegal (5.35%), South Africa (4.6%), and Thailand (3.4%) [3–7]. the prevalence of AF in our study is likely to be a valid estimate for the Tanzanians hypertensive population. The center attends patients from the entire country tertiary center in the country serving patients with cardiovascular diseases. The preponderance of AF and high BP seen in males compared with females in our study has been reported in other studies could be due to degenerative process of atrial muscle and conducting cell [8–12].

Alcohol consumption was common among study population (42.5%). Studies have shown that alcohol is known to produces arrythmogenic substrates thus triggering AF [13–15].

In our study more than half of the patients with AF were found to have low total cholesterol, triglyceride, HDL and high LDL, similar trend was seen in MESA, FHS and post hoc analysis of lipid lowering treatment to prevent heart attack trial (ALLHAT) [16, 17]. however a study from Japan showed high levels of cholesterol, HDL, LDL being associated with decreased risk of AF while triglyceride were not associated with AF [18].

The most common clinical presentation of participants with AF were palpitation and easy fatigability which were significantly higher compared to no AF group similar pattern was observed in Kenya at Aga Khan University Hospital, Nairobi looking at clinical characteristics and outcomes of atrial fibrillation and flutter [19].

More than half of the participants with AF had their systolic blood pressure 140 mmHg and normal pulse rate with majority of them on antihypertensive medication, most common groups of medication used were (60%) ACEI/ARB, (70%) diuretics and (55%) beta-blockers. As shown in the study patients with AF presented with symptoms of palpitation and are more likely to receive beta blocker in the combination of their anti-hypertensive medication thus rate controlled AF. In the current study it was also observed that atrial fibrillation was more common among those with dilated left atrial and ejection fraction < 50% which supports the pathogenesis theory of atrial fibrillation.

Results of our study have some clinical implications. Our prevalence estimate of AF 10.25% in hypertensive is high considering the chronic course of this disease that might cause serious thromboembolic stroke. Our study has demonstrated that there are other factors that could even further increase prevalence of AF for example, males older than 58 years of age with BP ≥140/80 mmHg. Awareness and education on regular monitoring and compliance may help reduce the number of serious strokes related to AF by giving stroke prophylaxis treatment.

This study was not short of limitations. For instance, owing to the cross section nature of this study both predictor and outcomes variable were measured simultaneously and thus our findings cannot infer causality. Furthermore, as this study was conducted in a tertiary level hospital, referral filter bias is probable. The strength of this study it was conducted at tertiary center where patients from all over Tanzania are referred for advanced management thus the sample is representative of the whole nation.

## Conclusion

Prevalence of AF in hypertensive population was found to be high. We recommend every hypertensive patient to have 12 lead ECG for early recognition of AF and prompt management.

## Supplementary information

**Additional file 1.**

**Additional file 2.**

## Data Availability

The final version of Data set supporting the findings of this paper is submitted together with this manuscript to the editorial committee. All the raw data is included in this manuscript.

## Abbreviations

AF: Atrial fibrillation
ECG: Electrocardiography
SBP: Systolic blood pressure
DBP: Diastolic blood pressure
LDL: Low density lipoprotein
HDL: High density lipoprotein
JKCI: Jakaya Kikwete cardiac institute
BP: Blood pressure
LAD: Left atrial diameter
BMI: Body mass index
EF: Ejection fraction
EGFR: Estimated glomerular filtration rate
OR: Odds ratio
ESC: European society of cardiology
SPSS: Statistical package for social sciences
ACEI: Angiotensin converting enzyme inhibitor
ARB: Angiotensin receptor blocker
CI: Confidence interval
LVH: Left ventricular hypertrophy
IVSD: Intraventricular septal defect
