# Supplementary material for: Burden and correlates of atrial fibrillation among hypertensive patients attending a tertiary hospital in Tanzania
Source: BMC Cardiovasc Disord. 2020 May 19;20:235. doi: 10.1186/s12872-020-01517-x (PMC7236463; doi:10.1186/s12872-020-01517-x)
Supplement: Supplementary file 2 — Additional file 2. [file 12872_2020_1517_MOESM2_ESM.docx]

**APPENDIX II QUESTIONNAIRE**

**Q No. ………………………………**

1. Are you Hypertensive?

1. No, thank you for your time

2. Yes, continue with no 2

1. When were you diagnosed?..............
2. < 1 month ago
3. 1-3 months ago
4. 3-6 months ago
5. 6months – 1 year
6. 1-3 years
7. >3yrs ago
8. Do you take medication?
9. No
10. Yes
11. **Reg no** ………………………………
12. Date of interview……………
13. **Contacts** of patient/ Next of Kin;……………………………
14. **Residence**……………………
15. Age…………………………
16. 18-27
17. 28-37
18. 38-47
19. 48-57
20. 58-67
21. SEX
22. MALE
23. FEMALE
24. Education;
25. Informal
26. Primary
27. Secondary
28. Higher education
29. Occupation;
30. Unemployed
31. Peasant/ petty trader
32. Employed
33. Self employed
34. Others…………
35. Marital status
36. Single
37. Married/ cohabiting
38. Divorced/widow
39. Blood pressure readings;

**BP1…………/………………**

**BP2**…………/………………

Ave BP …………/………….

1. Pulse………………
2. Tachycardia
3. Bradycardia
4. Normal
5. Pulse rhythm
6. Regular
7. Irregularly irregular
8. Regularly irregular
9. Temperature …………..
10. Normal
11. Fever
12. Respiration Rate………… ……..
13. Normal
14. Tachypnea
15. Bradypnea
16. **Weight ……………kg**
17. **Height** ……………..cm
18. BMI ………………….
19. Underweight
20. Normal
21. Overweight
22. Obese
23. What **symptoms** brought you to the hospital today?
24. Chest pain
25. Shortness of breath t
26. Awareness of heart beat
27. Easy fatigability
28. Fainting attack
29. Cough
30. Body swelling
31. Follow up / refill of drugs
32. Others; ……………
33. How long have you been suffering from this/ these symptoms?
34. Minutes
35. 1-24 hours
36. 1 day to 7 days
37. > 1 week
38. Have you ever been diagnosed with **Diabetes mellitus?**
39. No go to no 27
40. Yes
41. For how long have you been known to be diabetic?
42. Less than one month
43. 1-6 months
44. 6months-1 year
45. 1-5 yrs
46. How do you **control** your blood sugar level?
47. Regular oral medication
48. Oral medication when blood sugar is high
49. Regular insulin
50. Insulin when blood sugar is high
51. Both insulin and oral medication
52. Diet and / exercise
53. None
54. Have you ever been diagnosed with **dyslipidemia?**
55. No go to no. 30
56. Yes mon
57. When were you diagnosed with dyslipidemia?
58. < 6 months ago
59. 6m – 1 year ago
60. 1-5 years ago
61. >5 years ago
62. How do you **control** your dyslipidemia?
63. Regular medication
64. Diet and exercise
65. Herbal medication
66. Others ………….
67. Have you ever been diagnosed with **CKD**?
68. No go to no 33
69. Yes
70. When were you diagnosed with ckd?
71. 6<months ago
72. 6months – 1 year ago
73. 1-5 years ago
74. > 5 years ago
75. What **medication** are you **currently** on?
76. ACEI
77. ARB
78. Betablocker
79. Diuretics
80. Vasodilators
81. Calcium channel blocker
82. antiplatelet
83. Others:…………
84. Do you **smoke**?
85. No got to no 36
86. Past
87. Current
88. How long have you smoked for in your life?
89. <6 months
90. 6months to 1 year
91. 1-3 years
92. >5 years
93. How many sticks in average do you a day?
94. 1-5
95. 5-10
96. 1 pack
97. > 1 pack
98. Do you use **alcohol**?
99. No go to no 40
100. Used to
101. Yes
102. What **kind** of alcohol did/do you use?
103. Wine
104. Beer
105. Spirit
106. Local brew
107. Others …………….
108. How **often** do you use?
109. Occasionally
110. Once a month
111. Once a week
112. Twice/ thrice a week
113. Daily
114. How much do you drink a sitting?
115. 1-3 glasses
116. 1-3 bottleS
117. 4-7 bottles
118. 8-10 bottles
119. >10 bottles
120. 1 litre
121. Others

**ECHO, ECG and Biochemistry test results**

1. ECG
2. LVH
3. No LVH
4. Ventricular rate ………….
5. Below 50
6. 50- 99
7. >100
8. P wave on ECG
9. Present
10. Absent
11. R-R interval
12. Regular
13. Irregular
14. AF
15. Present
16. Absent
17. ECHO
18. IVSD ………………mm
19. LAD………………..mm
20. EF ……………………%
21. CONCLUSION……………………………………….
22. Serum Total cholesterol …………………………..MMOL/L
23. Serum HDL…………………………………….….……..MMOL/L
24. Serum LDL………………………………………..………MMOL/L
25. Serum Triglyceride …………………….…….………mg/dl
26. Serum Creatinine ……………………………….….Umol/l
27. RBG…………………………………………………..…...mmol/l
28. Hb …………………………………………………………..g/dl
29. MCV…………………………………………………………FL
30. MCHC……………………………………………………….G/DL
31. WBC……………………………………………………… X10^12^/L
32. PLT…………………………………………………………X10^9^/L
33. Uric acid ……………………………………………….MG/DL
34. CRP………………………………………………………..MG/L
35. ESR………………………………………………………..MM/HR
36. T3………………………………………………………….NG/ML
37. T4………………………………………………………….NG/ML
38. TSH………………………………………………………..NG/ML
